# Somnambulism: Diagnosis and treatment

**DOI:** 10.4103/0019-5545.33261

**Published:** 2007

**Authors:** Rahul Bharadwaj, Suresh Kumar

**Affiliations:** Department of Psychiatry, Post-Graduate Institute of Medical Education and Research, Chandigarh, India

**Keywords:** Epilepsy, somnambulism, treatment

## Abstract

Somnambulism is an arousal disorder that is usually benign, self-limited and only infrequently requires treatment. Chronic sleepwalking in children has been shown to be associated with behavioral problems and poor emotional regulation. Most cases can be diagnosed with careful noting of case history and epilepsy is an important differential diagnosis. Management with pharmacological and behavioural measures is usually safe and effective. We present two cases of somnambulism that highlight the importance of the diagnosis and treatment of this condition.

## INTRODUCTION

Somnambulism is an arousal parasomnia consisting of a series of complex behaviors that result in large movements in bed or walking during sleep. Most frequent in children (2-14% of children), it is usually a benign, self-limited maturational occurrence.[1] Sleepwalking often decreases with the onset of puberty, but at least 25% of children with recurrent sleepwalking may continue to sleepwalk in adulthood.[2] Chronic sleepwalking in children has been shown to be associated with other, often subtle, sleep disorders, behavioral problems and poor emotional regulation. In addition, recent evidence indicates that a good night's sleep plays a critical role in early brain development, learning and memory consolidation.

It has also been reported that parents of these children frequently do not pursue medical treatment due to either intermittent occurrence of these events despite medication or reluctance to having their child continuously receive psychotropic drugs.[2] Although some pediatric sleep disorders demand medical attention (*e.g.*, obstructive sleep apnea, narcolepsy), the majority require clinical assessment and intervention skills that specialists in behavioral medicine are ideally suited to provide.[3] We present here two cases of sleepwalking in children that highlight the importance of the differential diagnosis and treatment of this condition.

## CASE REPORTS

### Case 1

A male child aged 8 years, 10 months, a student of the fourth grade, hailing from a Hindu nuclear family of middle socio-economic status and an urban background presented to our center with the complaint that he had been walking in his sleep for the past five months. After going to bed at about 11 pm, he would get out of bed between 12 midnight and 3 am and perform a variety of activities—go to the toilet and urinate, recite pieces of conversation, act out a scene from a TV serial, etc. During this period, he would never attempt to leave the room and never injured himself. He would walk with his eyes open, but he did not respond to the calling out of his name.

Each episode would last for 5-7 minutes and would end with the patient returning to his bed and resuming his sleep. Only one episode would occur in a single night and these would occur once every 1-2 weeks. The patient had no recollection of the episode on waking up. In the month prior to his presentation at the clinic, the episodes had increased to once every alternate night. There was no history suggestive of any daytime interference in the scholastic and interpersonal functioning of the child. Past history was unremarkable and the child had attained normal developmental milestones. There was a family history of a psychotic illness in a cousin and alcohol dependence in a paternal uncle. There was no family history of sleep walking or epilepsy. No stressors could be identified and the mental status examination was unremarkable. Mean intelligence quotient (IQ) at the time of detailed assessment was 116.

A diagnosis of somnambulism (F51.3 as per International Classification of Diseases (ICD-10) criteria)[4] was made. An electroencephalogram (EEG) was done that showed evidence of sharp waves. A pediatric neurologist was consulted and EEGs were repeated —awake and sleep-deprived, which were normal. He was not prescribed any antiepileptic medication. He was put on Tab. Lorazepam 1 mg at bedtime and was advised to follow a consistent sleep schedule with which symptoms stopped within a month. The family was educated about the disorder and reassured. After three months, the medication was reduced at the rate of ½ mg a month and then stopped. An EEG was repeated and found normal. Symptoms had not recurred up to the last follow-up six months following the stoppage of medication.

### Case 2

A male child aged 13 years, 8 months, a student of the eighth grade, hailing from a Hindu extended family of middle socio-economic status and an urban background, presented to our center with the complaint that he had been walking in his sleep for the past three years. About a month prior to the onset of these symptoms, the patient had suffered a generalized tonic-clonic seizure. Computed tomography (CT) and magnetic resonance imaging (MRI) scans and EEG done at that time had been normal. He received Carbamazepine 300 mg/day and Phenobarbitone 50 mg/day for one week. Medications were stopped when investigations were found to be normal. The seizure did not recur. About the same time, the patient lost his grandfather to whom he was much attached.

The episodes of walking in his sleep would usually occur in the first four hours of sleep in the night but had never occurred in the day. During these episodes, the patient would walk about the room or recite lessons learned in the day. Each such episode would last for up to half an hour and any attempt made to communicate with him during this period would be unsuccessful. The patient had no recollection of the episode on waking up. Over three years, the frequency of episodes had gradually increased to about thrice or four times per week. Over the same period, the patient had begun performing poorly in science and social studies. He would experience frequent and severe headaches (not suggestive of migraine) that would interfere with his studies.

At the time of consultation, scholastic difficulties had also worsened with the patient having failed in the 8^th^ grade. He was under considerable pressure from his parents to improve academically. They were worried that there was “something wrong with his brain.” The patient had been born preterm (at ≈ 33 weeks of gestation) and had a low birth weight. Motor and language milestones had developed normally. There was a family history of mental retardation in the elder sister and of seizure disorder in mother. There was no family history of somnambulism. Physical examination was normal and a mental status examination was unremarkable. IQ was 84 at initial assessment.

A diagnosis of somnambulism (F51.3 as per the ICD-10 criteria)[4] was made. Sleep-deprived EEG and a noncontrast CT head were done, which were normal. The parents were educated about the patient's illness, reassured, advised to reduce their pressure on the patient and be supportive. In addition, he was advised to follow a consistent sleep schedule, avoid sleep deprivation and was prescribed 1 mg lorazepam at bedtime. Over the next two months, the somnambulism episodes stopped completely and lorazepam was reduced to ½ mg for the next one month and then stopped. Over the next six months, he improved scholastically with support from his parents and additional coaching with his studies. His headaches stopped occurring. He was followed up and was found symptom-free one year later.

## DISCUSSION

Somnambulism is a disorder of arousal. It generally occurs in deep nonrapid eye movement (NREM) (stages 3 and 4) sleep and, thus, most often takes place in the first third of the night when these sleep stages are most common. The event may last from several minutes to an hour. There is complete amnesia about the event after waking up from sleep. Somnambulism can consist of very complex motor activity (as in case 1) of which walking is just one element. Somnambulism may be precipitated by a variety of conditions such as insufficient sleep resulting from an irregular sleep schedule, staying up late, giving up a daily nap or waking early in the morning.[3]

Other factors implicated are fever, stress (operative in case 2), medications (phenothiazines, chloral hydrate, lithium) and other disorders that result in arousals (obstructive sleep apnea, distended bladder, loud noise).[1] Several recent studies report no clear association between psychopathology and somnambulism.[5] Diagnosis is easily established by history alone. In some cases of nontypical history with very complex stereotypical automatism or unusual behaviors, seizures (especially frontal or temporal lobe epilepsy or partial complex seizures) should be suspected. An EEG is indicated in these cases. In the cases reported here, the behaviors were neither suggestive of automatisms nor atypical for somnambulism.

Although electrical discharges in the EEG could be suggestive of some sort of epilepsy, as in the initial EEG in case one, EEG abnormalities have been reported in up to 47% of patients with parasomnias.[6,7] In addition, ‘slow wave activity’ in EEG in patients with somnambulism has been reported to persist to an older age. This is in keeping with the theory that arousal disorder is associated with delayed maturation of the central nervous system (CNS). Thus, while it is essential to rule out a diagnosis of epilepsy, it is also important not to be over-inclusive while diagnosing it. Sensitivity to nonepileptic EEG activity in patients with parasomnias helps overt this.

In the second case, episodes of somnambulism began one month after the patient had lost his grandfather and after he had experienced a grand mal seizure. EEG records and neuroimaging done on two occasions were normal. Epileptic patients with partial seizures and those with seizures are more frequently at risk for developing sleep disorders. Primary or secondarily generalized tonic-clonic seizures reduce total sleep time, reduce the proportion of rapid eye movement (REM) sleep and increase the proportion of NREM sleep time.[8] Since our patient (case 2) had only one grand mal seizure, we can only speculate that these mechanisms were operative in this case at the onset of somnambulism.[9] Other factors like prominent psychological stress were also present in this otherwise vulnerable child. It is likely therefore, that in this case, a complex interaction of these factors was etiologically related to the sleep disorder.

Treatment is considered when the frequency of events is high (as in both the cases reported), psychosocial complications or stressors are present (as in case 2) or when events are violent and potentially injurious. A low-dose benzodiazepine is the drug of choice (lorazepam was given in both the cases), although tricyclic antidepressants and trazodone may be beneficial as well.[1] In addition, behavioral management in the form of scheduled awakenings and a positive bedtime routine is essential. We found adherence to a positive bedtime routine beneficial in both the reported cases. The mechanism of this intervention in decreasing sleepwalking in the cases we report is unclear. One possibility is that the positive routine altered the child's sleep patterns to eliminate the disruption in slow-wave sleep.

It could also be that the positive routine indirectly reduced arousal events by increasing the child's total sleep time.[3] In addition to these behavioural measures, appropriate interventions to manage psychosocial stressors are essential. In both the above cases, the episodes of sleepwalking had taken a progressive course and it is likely that without appropriate intervention, symptoms would have either persisted or progressed with adverse consequences for the child's intellectual and emotional development. These cases demonstrate the benefits and the need for the timely and appropriate intervention of this condition.
